# ITIH5-Derived Polypeptides Covering the VIT Domain Suppress the Growth of Human Cancer Cells In Vitro

**DOI:** 10.3390/cancers14030488

**Published:** 2022-01-19

**Authors:** Michael Rose, Sebastian Huth, Marc Wiesehöfer, Josef Ehling, Corinna Henkel, Julia Steitz, Twan Lammers, Jennifer Kistermann, Oliver Klaas, Maximilian Koch, Sandra Rushrush, Ruth Knüchel, Edgar Dahl

**Affiliations:** 1 Institute of Pathology, RWTH Aachen University, 52074 Aachen, Germany; shuth@ukaachen.de (S.H.); marc.wiesehoefer@uk-essen.de (M.W.); Corinna.Henkel@bruker.com (C.H.); jenny.kistermann@googlemail.com (J.K.); Oliver.Klaas@ukmuenster.de (O.K.); maximilian.koch@uk-koeln.de (M.K.); sandrarushrush@hotmail.com (S.R.); rknuechel-clarke@ukaachen.de (R.K.); 2 Center for Integrated Oncology Aachen Bonn Cologne Duesseldorf (CIO ABCD), 52074 Aachen, Germany; 3 Department of Dermatology and Allergology, RWTH Aachen University, 52074 Aachen, Germany; 4 Department of Nanomedicine and Theranostics, Institute for Experimental Molecular Imaging, Uniklinik RWTH Aachen and Helmholtz Institute for Biomedical Engineering, RWTH Aachen University, 52074 Aachen, Germany; josef.ehling@gmx.de (J.E.); tlammers@ukaachen.de (T.L.); 5 Bruker Daltonik GmbH, 28359 Bremen, Germany; 6 Institute for Laboratory Animal Science, University Hospital, RWTH Aachen University, 52074 Aachen, Germany; jsteitz@ukaachen.de

**Keywords:** breast cancer, bladder cancer, lung cancer, ITIH5, tumor suppressor, vault protein inter-alpha-trypsin (VIT), tumor growth, biologicals

## Abstract

**Simple Summary:**

ITIH5 has been shown to be an effective tumor and metastasis suppressor which is being lost in various tumor entities including breast, bladder and lung cancer. In the present study a translational approach was pursued to develop truncated polypeptides derived from the full-length ITIH5 protein that are able to mimic its tumor suppressive functions. In fact, it was found that ITIH5´s vault protein inter-alpha- trypsin (VIT) domain (approximately 125 amino acids long) can specifically impair the growth of human cancer cells. Thus, our findings highlight polypeptides covering the VIT domain as a basis for the development of novel biological drugs potentially able to reactivate ITIH5 specific tumor-suppressive pathways in human cancers.

**Abstract:**

Oncogenic drivers such as mutated *EGFR* are the preferred targets in modern drug development. However, restoring the lost function of tumor suppressor proteins could also be a valid approach to combatting cancer. ITIH5 has been revealed as a potent metastasis suppressor in both breast and pancreatic cancer. Here, we show that ITIH5 overexpression in MDA-MB-231 breast cancer cells can also locally suppress tumor growth by 85%, when transplanted into the mammary fat pad of nude mice. For a potential drug development approach, we further aimed to define downsized ITIH5 polypeptides that still are capable of mediating growth inhibitory effects. By cloning truncated and His-tagged ITIH5 fragments, we synthesized two recombinant *N*-terminal polypeptides (ITIH5^681aa^ and ITIH5^161aa^), both covering the ITI heavy chain specific “vault protein inter-alpha-trypsin” (VIT) domain. Truncated ITIH5 variants caused dose-dependent cell growth inhibition by up to 50% when applied to various cancer cell lines (e.g., MDA-MB-231, SCaBER, A549) reflecting breast, bladder and lung cancer in vitro. Thus, our data suggest the substantial role of the ITIH5-specific VIT domain in ITIH5-mediated suppression of tumor cell proliferation. As extracellularly administered ITIH5 peptides mimic the growth-inhibitory effects of the full-length ITIH5 tumor suppressor protein, they may constitute the basis for developing anticancer drugs in the future.

## 1. Introduction

Inter-alpha-trypsin inhibitor heavy chain 5 (ITIH5) was identified as the fifth heavy chain member of the ITI family in 2004 [1]. Subsequently, *ITIH5* has been characterized as a putative tumor and metastasis suppressor gene in various tumor entities such as breast, bladder, lung, colorectal, and cervical cancer [2,3,4,5,6]. Loss of ITIH5 expression is mainly caused by DNA hypermethylation of the corresponding promoter regions and is correlated with unfavorable patient outcomes [2,3,4,5,6]. In turn, overexpression of ITIH5 has been shown to block cancer cell growth, migration and invasion in vitro [3,4,7]. In highly invasive human breast cancer cells of the triple negative phenotype (TNBC), ITIH5 expression triggered an epigenetic reprogramming associated with a shift in cell differentiation, which effectively suppressed the growth of metastases in vivo [7]. It has also been shown that ITIH5 may modulate the TGF-β signaling cascade via regulating the expression of the co-receptor endoglin, which antagonizes the inhibitor of differentiation 1 (Id1) [8]. Recently, p53 has been revealed as a putative transcriptional activator of *ITIH5* expression in melanoma that caused the inhibition of cell proliferation and metastasis [9]. Furthermore, by applying a genome-wide screen, Sasaki and colleagues identified *ITIH5* as one of the few candidate genes that can strongly suppress pancreatic cancer metastasis [10]. ITIH5 impaired metastasis formation in pancreatic cancer cells in vivo but it did not affect their growth rates. In pancreatic cancer stem cells, the long non-coding RNA (lncRNA) LINC00261 was suggested to bind to GATA6, increasing its activity at the *ITIH5* promoter [11]. These authors concluded that the presence of LINC00261 and GATA6 may impair the self-renewal and tumorigenesis capacity of pancreatic cancer stem cells, whereas the suppression of ITIH5 expression caused by LINC00261 downregulation rescued these tumorigenic functions.

Regulation of ITIH5 transcription and the prognostic and functional consequences of its silencing by promoter DNA methylation have been intensively studied in recent years, whereas the mode of action of the ITIH5 protein itself is still poorly understood. Numerous studies have suggested the hyaluronic acid (HA)-associated function of ITIH5 [12,13], namely that, analogous to other members of the ITI heavy chain family, a precursor protein is thought to be trimmed at a conserved cleavage site, unmasking a C-terminal amino acid involved in covalently binding HA [14]. As a consequence, ITIH5 could generate HA-fibrils (“cable-like structures”) similar to ITIH 1–3, stabilizing the extra cellular matrix (ECM) structures while affecting the binding characteristics of the HA receptor CD44 [15,16,17]. Martin and colleagues have shown that ITIH5 can cause stabilization of HA, which drives a TGF-β1-driven differentiation of fibroblasts to myofibroblasts associated with the HA-dependent co-localization of CD44 and epidermal growth factor receptor (EGFR) [12]. Recently, Huth et al. proposed the formation of HA cable-like structures by ITIH5 in the skin [13]. Additionally, the prognostic significance of ITIH5 has been demonstrated in bladder cancer in close association with abundant CD44 expression; matching this observation, SCaBER bladder cancer cells abundantly expressing CD44 could be sensitized to cisplatin treatment after forced ITIH5 expression in vitro [18].

However, the role of the family-specific domains i.e., the Vault protein inter-alpha-trypsin domain (VIT) and the Von Willebrand factor type A domain (vWA) [19], which are generally secreted to the ECM, and their contribution to tumor (growth) suppression is thus far completely unknown. In the present study, we aimed to give an insight into the ITIH5-mediated inhibition of tumor cell growth in a mouse model while considering the role of truncated ITIH5 proteins, with a special focus on VIT, independently of the HA-binding function. Since Weidle and colleagues have proposed to use ITI-derived proteins for the development of new anticancer drugs [20], here, we present ITIH5-derived polypeptides that mediate growth-inhibitory effects when simply administered extracellularly in vitro. This way, it may be possible to restore the tumor-suppressive functions of ITIH5 in cancer cells of various tumor entities that have lost their endogenous ITIH5 expression during tumor progression.

## 2. Results

### 2.1. Full-Length ITIH5 Inhibited the Cell Growth of TNBC Breast Cancer Cells In Vivo

**Figure 1:**
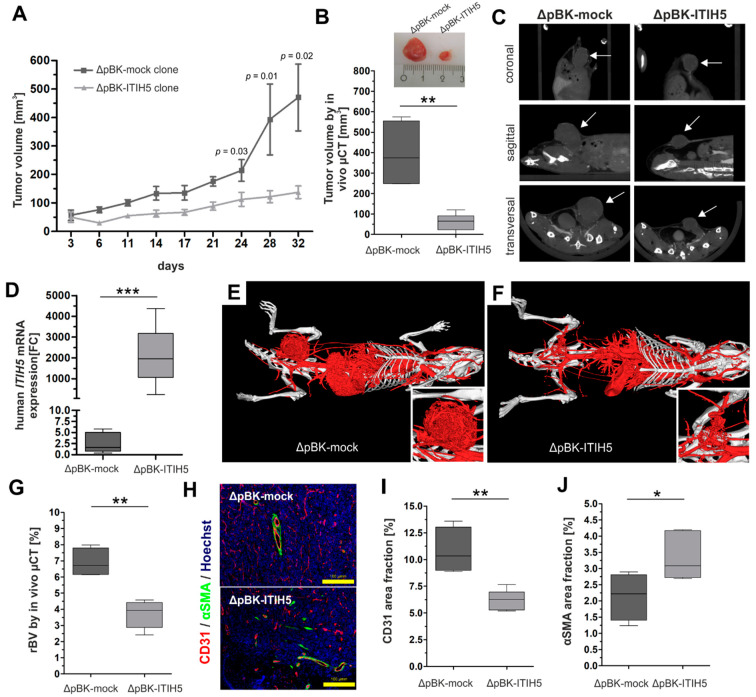
Full-length ITIH5 suppressed tumor cell growth in vivo. A tumorigenesis model was established to analyze the tumor growth of breast cancer cells either overexpressing ITIH5 or lacking ITIH5 expression and transplanted into the mammary fat pad. Tumor volume was determined by both using a caliper (**A**) and µCT imaging (**B**,**C** (see arrows)), showing the significantly decreased tumor growth of MDA-MB-231 breast cancer cells transfected with ITIH5. (**D**) ITIH5 overexpression in tumors grown in the mouse model was confirmed by qPCR analysis. In order to assess the putative effects of ITIH5 on the mechanisms of neovascularization, which may impact growth rates in vivo, we visualized the vascular system by contrast-enhanced µCT imaging (**E**,**F**) and calculated the relative blood volume (rBV) (**G**) between both groups. (**H**) Immunofluorescence staining of the markers involved in blood vessel maturation, i.e., CD31 (a specific marker for vascular structures) and αSMA (a specific marker for vascular differentiation). Hoechst dye was used to stain the nuclei. (**I**) The staining intensity of CD31 (**H**) was analyzed for tumors derived from either ΔpBK-mock (dark grey) or ΔpBK-ITIH5 clones (grey), shown as the area fraction. (**J**) The correlation analysis between relative blood volume (rBV) determined by µCT and the CD31 area fractions showed a significant association in the tumors of both groups. * *p* ≤ 0.05; ** *p* < 0.01; *** *p* < 0.001.

*ITIH5* was first revealed as a putative tumor suppressor gene in 2008 [7] and various studies confirmed the involvement of ITIH5 in impairing tumor cell growth in vitro [2,3,4,5,6,7,8]. In breast cancer, we demonstrated the ITIH5-mediated suppression of metastasis growth in vivo [7]. The in vivo effects of ITIH5 on breast cancer cell growth were still elusive. Therefore, we constructed a xenograft mouse tumorigenesis model through orthotopic transplantation of human breast cancer cells either overexpressing ITIH5 (ΔpBK-ITIH5 clone #12) or lacking ITIH5 expression (ΔpBK-mock clone #1) [7] into the mammary fat pad of BALB/c^nu/nu^ mice. Overall, *n* = 18 mice were included, i.e., nine mice per group. Tumor growth was measured and documented over 32 days by using a caliper. Beginning on Day 6 after tumor cell injection, the transplanted ΔpBK-mock control cells lacking ITIH5 expression grew much faster than the corresponding ΔpBK-ITIH5 cells showing ITIH5 overexpression (Figure 1A). 

After 32 days, the tumor volume (mm^3^) was finally determined by using µCT imaging, enabling comprehensive imaging of the 3D nodules. This non-invasive method confirmed the significantly decreased tumor growth of ΔpBK-ITIH5 clones (mean volume: 60.07; 95% lower–upper CI: 16.48–103.7) compared with ΔpBK-mock controls (mean volume: 393.0; 95% lower–upper CI: 125.4–660.5) by up to 84.7% on average (Figure 1B). Representative CT images showed induced tumors in the transverse, coronal and sagittal planes (Figure 1C). The ITIH5 expression status of the removed tumors derived either from ΔpBK-mock clones or ITIH5-overexpressing MDA-MB-231 cells was confirmed by qPCR (Figure 1D). Since tumors require oxygen and nutrients for their growth, in vivo tumor growth depends not only on proliferative characteristics but also on the ability to induce angiogenesis. Contrast-enhanced µCT (iodine-containing blood pool contrast agent: eXIA 1600r (Binitio Biomedical, Ottawa, ON, Canada)) was performed to visualize very small vessels up to 40 µm in diameter [21]. The CT images revealed a less developed blood supply in ΔpBK-ITIH5 induced tumors compared with ΔpBK-mock tumors, which showed a homogenous vascularization (Figure 1E,F). This notion was confirmed by calculating the relative blood volume (rBV) of the tumors grown in vivo. ΔpBK-ITIH5-induced tumors showed a highly significant reduction in relative blood volume compared with ΔpBK-mock tumors (Figure 1G).

Fluorescence staining of the endothelial cell marker platelet endothelial cell adhesion molecule-1 (PECAM-1, also referred to as cluster of differentiation 31 (CD31)), as well as the pericyte marker α-smooth muscle actin (αSMA) was performed to provide information about the density and distribution of blood vessels (CD31) and the maturity of blood vessels (αSMA) [21,22]. The overall degree of vessel maturation was significantly higher in ΔpBK-ITIH5 tumors (αSMA, *p* < 0.05). The ΔpBK-mock tumors showed significantly increased CD31 expression compared with the ΔpBK-ITIH5 tumors (Figure 1H,I). Thus, the differences in blood vessel density and maturation between ΔpBK-ITIH5 and ΔpBK-mock induced tumors are consistent with their differences in tumor growth: Slow-growing tumors such as ΔpBK-ITIH5 are characterized by a reduced density of blood vessels. In contrast, fast-growing tumors such as ΔpBK-mock tumors form a dense network of blood vessels that are immature and small-lumened. Given these angiogenic differences, the rBV and amount of CD31 showed a positive correlation (r^2^: 0.83, *p* < 0.001) and we confirmed that in ΔpBK-ITIH5 induced tumors, the decreased relative blood volume was associated with decreased CD31 staining.

### 2.2. ITIH5 Protein Downsized to the Secreted N-Terminal Part without the HA-Binding Function Inhibited Cancer Cell and Colony Growth In Vitro

**Figure 2:**
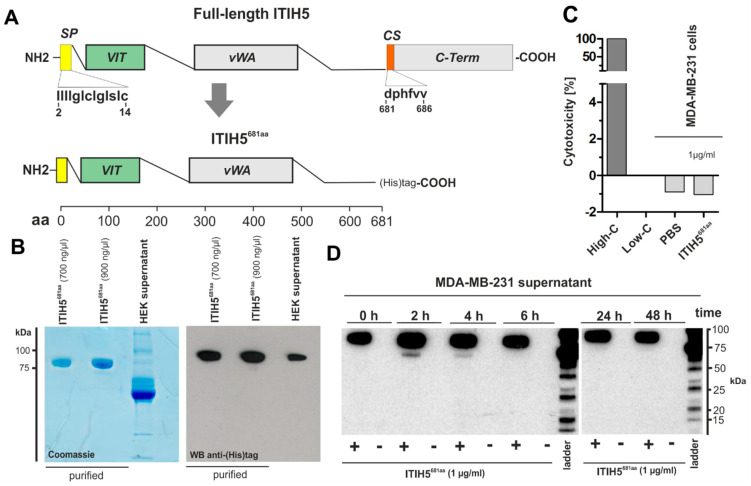
In Vitro synthesis, toxicity, stability, and specificity of recombinant *N*-terminal ITIH5t polypeptides. (**A**) Schematic drawing of the full-length ITIH5 protein (942 aa) and the derived *N*-terminal ITIH5t protein (681 aa), which includes both the VIT (relative aa position according to specific databases: PFAM = 51–159; Prosite profiles = 35–161) and vWA (relative aa position: PFAM = 295–466; Prosite profiles = 295–478) domains. (**B**) Visualization of the recombinantly produced *N*-terminal ITIH5t polypeptide after purification by using Coomassie staining and Western blotting. Immunodetection was performed using a His-tag antibody. (**C**) A LDH cytotoxicity assay was performed. The recombinant *N*-terminal ITIH5t did not show any toxicity. (**D**) The biochemical stability of *N*-terminal ITIH5t applied to the supernatant of aggressive MDA-MB-231 breast cancer cells showed an undiminished peptide concentration, which could be re-isolated over a period of 48 h.

The mode of action of ITIH5 protein, besides its HA-binding function, is not known. In particular, the roles of the ITI heavy chain specific protein domains are still poorly understood. In the following, we focused on the secreted protein domains, particularly on VIT. We constructed truncated ITIH5t polypeptides covering the signal peptide responsible for secretion of the VIT and vWA domain but excluding the cleavage site which is required for covalent HA binding (Figure 2A). cDNA sequences were cloned into a CMV containing the expression vector construct (pMS-L-A-IV) including an in-frame C-terminal His-tag required for purification and protein verification. The pMS-L-A-IV-N-term-ITIH5t vector was further used for transfection of HEK293-T cells. *N*-terminal ITIH5t proteins were subsequently isolated from the supernatant of transfected HEK cells using an Ni-NTA agarose-based affinity chromatography matrix purifying recombinant ITIH5t proteins carrying the His-tag. *N*-terminal ITIH5t proteins (subsequently named ITIH5^681aa^) that had been rebuffered in PBS and enriched via Vivaspin 6 columns (filtering out proteins ≤ 50 kDa) were confirmed by Coomassie staining and Western blot analysis (Figure 2B), followed by mass spectrometry analysis (Figure S1). The band detected in the 2D gel fits the expected molecular weight of ~75 kDa and the isoelectric point (IP) calculated in silico with a pH of 8.96, which, however, may vary (both IP and molecular weight) due to different post-translational modifications [23]. A mass spectrometric PMF (peptide mass fingerprint) analysis of the spots confirmed the detection of ITIH5 in the supernatant of human cells with high statistical accuracy (score range of the different spots: 67–197).

Subsequently, purified and confirmed ITIH5^681aa^ was applied to breast cancer cells in vitro. We did not observe the unspecific cytotoxicity (necrotic impact on cell membranes) of recombinant *N*-terminal ITIH5t proteins (Figure 2C). ITIH5^681aa^ was also quite stable, since it could be re-isolated without a significant loss of concentration after its application to the environment of aggressive breast cancer cells (MDA-MB-231) over a period of at least 48 h (Figure 2D).

**Figure 3:**
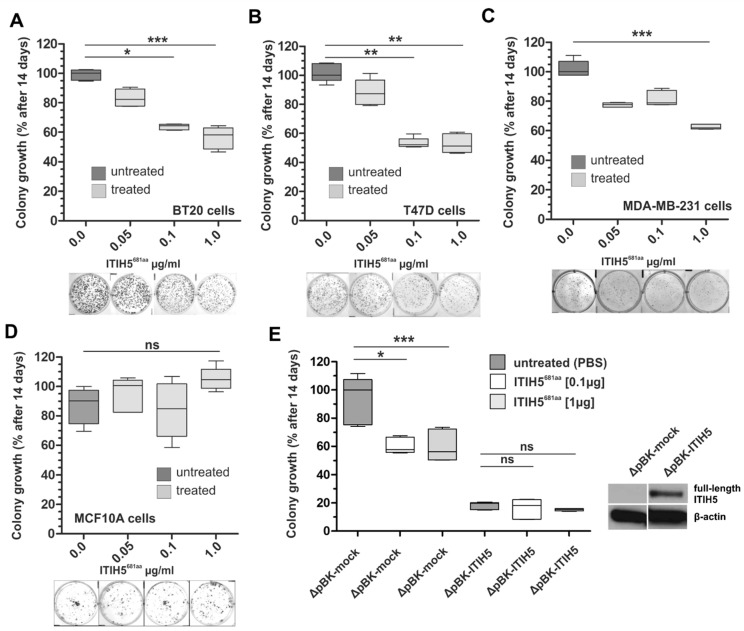
*N*-terminal ITIH5t protein (ITIH5^681aa^) suppressed the colony growth of malignant breast cancer cell lines but not of the benign breast cell line MFC10A. (**A**–**D**) A clonogenic survival assay was performed under *N*-terminal ITIH5t application over 14 days. The dose-dependent suppressive effect of *N*-terminal ITIH5t on colony formation is shown as a box plot and by representative growth colonies (below graphs) for (**A**) basal BT20 (**B**) luminal T47D and (**C**) basal MDA-MB-231 breast cancer cells. (**D**) The suppressive impact of *N*-terminal ITIH5t on benign MCF10A breast cancer cells was not observed. * *p* ≤ 0.05; ** *p* ≤ 0.01; *** *p* ≤ 0.001; ns: not significant (*p* > 0.05). (**E**) Based on in vitro models of breast cancer cells stably re-expressing ITIH5 (ΔpBK-ITIH5) and the corresponding mock clones (ΔpBK-mock) (see also Figure 1 and Rose et al. [7]), clonogenic survival assays were performed under *N*-terminal ITIH5t application (0.1 and 1.0 µg/mL) to assess the specificity. Only mock control clones lacking ITIH5 expression were significantly responsible for the suppressive effects mediated by the applied *N*-terminal ITIH5t. ΔpBK-ITIH5 clones overexpressing the full-length ITIH5 protein were not further affected by the *N*-terminal ITIH5t treatment. Note that growth differences between the mock clones (ΔpBK-mock) treated with ITIH5t and the overexpressing clones (ΔpBK-ITIH5) could be caused by various variables, including the different exposure times of the administered vs. endogenously expressed proteins.

Next, a clonogenic survival assay was performed for ITIH5^681aa^ treatment using various malignant breast cancer cell lines, i.e., basal A BT20 cells, luminal T47D cells and basal B MDA-MB-231 cells (Figure 3A–C). In all three cell lines, the dose-dependent impact of ITIH5^681aa^ on colony growth was observed, which varied between 39% and 50% growth inhibition when 1 µg/mL ITIH5^681aa^ was applied. ITIH5^681aa^ was renewed every 72 h. The benign cell line MCF10A did not significantly respond to ITIH5^681aa^ treatment and thus no growth reduction was observed (Figure 3D). Using the previously generated stable MDA-MB-231 clones either lacking (ΔpBK-mock clone #1) or overexpressing ITIH5 (ΔpBK-ITIH5 clone #12) ([7]), we further confirmed the impaired colony growth in mock control cells only under ITIH5^681aa^ treatment (median Δgrowth at 1 µg/mL between untreated and treated cells: 43.7%). MDA-MB-231 cells stably expressing full-length ITIH5 were not significantly affected by the application of ITIH5^681aa^ (median Δgrowth at 1 µg/mL between untreated and treated cells: 4.4%), supporting the concept that growth inhibition by ITIH5^681aa^ occurs in an ITIH5 signaling-specific context, i.e., ITIH5^681aa^ mimicked the full-length ITIH5’s function by targeting similar pathways/factors, while the ITIH5 signal was already affected/saturated in the ΔpBK-ITIH5 clone by endogenously expressed full-length ITIH5 protein, which could not be triggered further.

### 2.3. ITIH5 Downsized to the VIT Domain Was Sufficient and Responsible for Cancer Cell Growth Inhibition, Similar to the N-Terminal Protein

Finally, we downsized the ITIH5 protein to the VIT domain including the signal peptide (amino acids 1–161), subsequently named ITIH5^161aa^. Alignment by using compositionally adjusted substitution matrices [24,25] of the ITIH5-derived VIT with full-length human ITIH1-4 protein sequences revealed only slight sequence identity, varying between 26% and 37% at relative amino acid positions between 51–161 relative to the ITIH5 protein (Table 1). There was no significant sequence identity of ITIH5^161aa^ for amino acid positions 1–50 when aligned with ITIH1-4.

**Figure 4:**
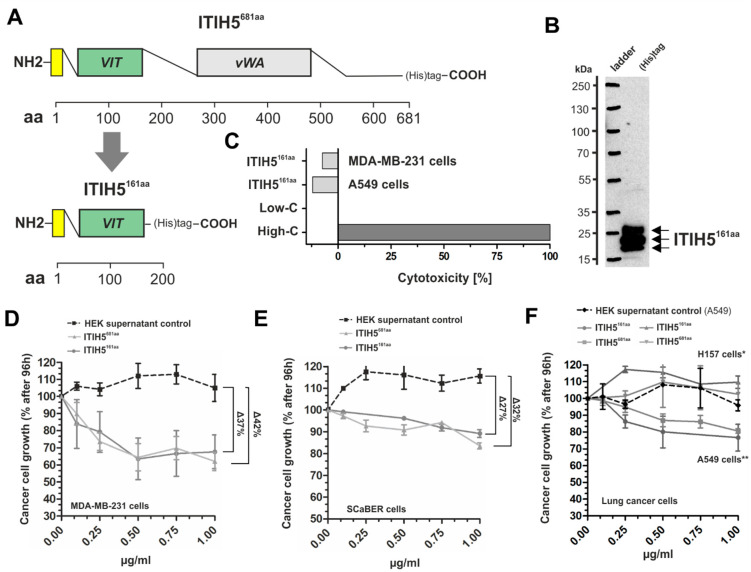
Tumor-suppressive impact of the recombinant VIT-domain containing polypeptide (ITIH5^161aa^) derived from the *N*-terminal ITIH5t protein applied to breast, bladder and lung cancer cells. (**A**) Schematic drawing of the *N*-terminal ITIH5^681aa^ polypeptide (681 aa) and the truncated ITIH5^161aa^ polypeptide (161 aa) covering the VIT domain. (**B**) Visualization of the recombinant ITIH5^161aa^ produced after purification using immuno-detection via a His-tagged antibody. (**C**) A LDH cytotoxicity assay was performed using breast and lung cancer cells. The recombinant ITIH5^161aa^ did not show any non-specific cytotoxicity. (**D**–**F**) In vitro dose–response curve analysis confirmed the concentration-dependent growth inhibition (27–42%) that was similar to treatment with either ITIH5^681aa^ or ITIH5^161aa^ polypetides for 96 h in (**D**) aggressive breast cancer cells (MDA-MB-231) and (**E**) squamous basal bladder cancer cells (SCaBER). (**F**) Specific growth inhibition was confirmed in lung adenocarcinoma cells (A549), whereas squamous lung cancer cells (H157) did not respond to either of the truncated ITIH5 polypeptides. **Note:** Purified scrambled proteins (processed in a similar manner to the truncated ITIH5 proteins) of the supernatant of the host HEK cells originally used for in vitro production of the recombinant truncated ITIH5 proteins showed no growth-inhibitory effects. Treated cells were normalized to the scrambled controls, since HEK proteins increase cell growth rates.

**Figure 5:**
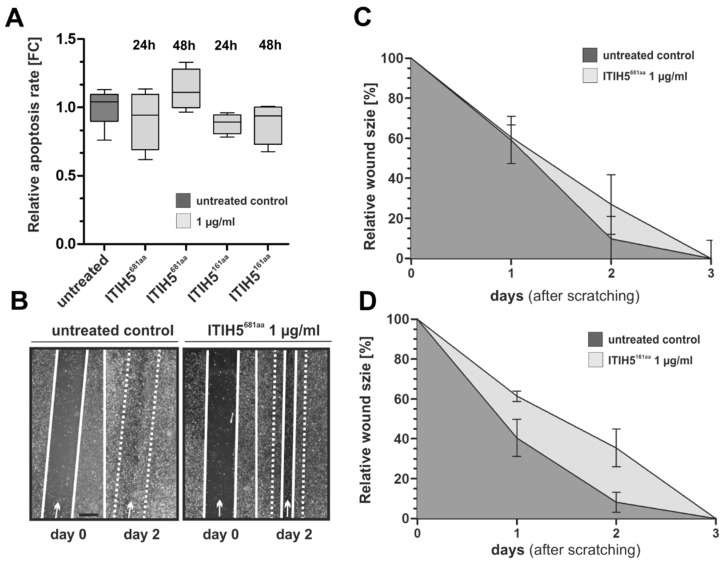
The impact of ITIH5^681aa^ and ITIH5^161aa^ treatment on the apoptosis and motile characteristics of MDA-MB-231 breast cancer cells. (**A**) Caspase 3/7 activity as an indicator of apoptosis in MDA-MB-231 cells either treated with ITIH5^681aa (^1 µg/mL) or ITIH5^161aa^ (1 µg/mL) for 24 h and 48 h. The box plot shows the relative apoptosis rate. Horizontal lines: grouped medians. Boxes: 25–75% quartiles. Vertical lines: range, minimum and maximum. (**B**–**D**) Cell migration was analyzed by using a wound healing assay. (**B**) Representative wound area documentation by light microscopy 0 and 48 h after scratching. White line: cell-free wound area. White dashed line: original wound area size at 0 h. Original magnification 50×. (**C**,**D**) The mean migration rate of an untreated (PBS) control cell set and MDA-MB-231 cells treated with either (**C**) ITIH5681aa (1 µg/mL) or (**D**) ITIH5161aa (1 µg/mL) was analyzed over 3 days. Vertical lines: standard deviation (S.D.) of triplicates. The cell-free area on Day 0 was set as 100% and used for standardization.

The synthesis of ITIH5^161aa^ was analogous to ITIH5^681aa^ using a cloned vector construct comprising VIT cDNA, including a *C*-terminal His-tag (Figure 4A), which was used for transfection of HEK293 T cells. Isolated and rebuffered ITIH5^161aa^ polypeptides were confirmed by Western blotting (Figure 4B). Interestingly, all batches of purified ITIH5^161aa^ polypeptides showed a pattern of three distinct bands close to the predicted molecular weight of the ITIH5^161aa^ polypeptides (~18.3 kDa; online Peptide and Protein Molecular Weight Calculator) which may suggest extensive post-translational modifications similar to those of the ITIH5^681aa^ protein as visualized in the 2D gel. Protein enrichment was achieved by filtering out contaminating proteins ≤ 10 kDa. In vitro application of recombinant ITIH5^161aa^ to breast (MDA-MB-231) and lung cancer cells (A549) did not cause any necrotic effects in a cytotoxicity assay (Figure 4C). In turn, drug response assays revealed the dose-dependent impairment of cell growth under ITIH5^161aa^ treatment over 96 h in breast (MDA-MB-231), bladder (SCaBER) and lung cancer cells (A549). The dose-dependent curves were highly similar (±5%) to the growth inhibition rates mediated by ITIH5^681aa^ application (Figure 4D–F), including the lack of an impact on squamous-type H157 lung cancer cells (Figure 4F). Scrambled proteins of the supernatant of wild-type HEK cells which had been isolated and rebuffered in a similar manner to the truncated ITIH5 proteins were used as control to exclude (unspecific) growth-inhibitory effects potentially mediated by HEK-derived proteins (≥10 kDa). Compared with the control, both ITIH5^161aa^ and ITIH5^681aa^ proteins caused growth inhibition by up to ~40%. We did not determine any significant changes in apoptosis under ITIH5 application (Figure 5A) in breast cancer cells 24 and 48 h after treatment.

Finally, we performed migration studies based on a wound healing assay to give some initial insights into whether truncated ITIH5 polypeptides could also impair the progressive features of cancer cells. The data are shown in Figure 5B–D and may support a putative effect on motility capabilities, which, however, does not clearly seem to be a general effect, since only the ITIH5^161aa^ treatment (1 µg/mL) mediated reduced wound seeding by 24 h after scratching (Figure 5D).

## 3. Discussion

The treatment success of new anticancer therapies depends, to a large extent, on the particular tumor entity, the classified histological or intrinsic subclass, and the individually associated molecular characteristics. In breast cancer, for instance, patients with a luminal and estrogen (ER)-positive tumor may respond to antihormonal therapies [26,27], whereas effective treatment is still lacking for patients with triple-negative breast cancers (TNBC) [28]. Recently, immune checkpoint inhibitors targeting immune escape have revolutionized treatment options for various cancer entities (e.g., bladder cancer [29]), and may also offer a promising approach to treating TNBC [30,31]. However, as seen in bladder cancer, objective response rates of up to only 30% demonstrated that only some of the patients benefit [32], and overall outcomes have improved marginally [33]. Therefore, new approaches are still needed to find attackable vulnerabilities for many cancer entities or cancer subgroups to further improve patient outcomes in the future. By taking advantage of the vast amount of molecular information available nowadays for defined cancer subtypes (e.g., on the expression, mutation, methylation and pathway components), we can, for example, start a new approach to evaluate the clinical efficacy of reactivating tumor suppressor genes or their pathways. *ITIH5* is a typical so-called Class II tumor suppressor gene [34] that is inactivated by promoter DNA methylation rather than by genetic alteration [2,3,4]. In recent years, the functional consequences of the loss of ITIH5 expression have been intensively studied, highlighting its important role as both a tumor and metastasis suppressor gene in various tumor entities [2,3,4,5,6,7,8,9,10]. So far, the metastasis-inhibitory properties of ITIH5 have been extensively investigated in vivo [7,10,35]. In this study, we present in vivo data indicating that ITIH5 can also locally and strongly suppress tumor growth in breast cancer. This prompted us to further investigate whether truncated ITIH5 proteins applied extracellularly to tumor cells could suppress tumor growth in various cancer entities. The positive results at relatively low peptide concentrations indicate that truncated ITIH5 proteins could be prospectively developed as biologicals that could be useful in TNBC therapy, for example.

### 3.1. Animal Model Showing ITIH5-Mediated Tumor Cell Growth Inhibition

In the present study, we confirmed ITIH5-mediated tumor cell growth inhibition in vivo. Breast cancer cells overexpressing the full-length ITIH5 and transplanted into the mammary fat pad of mice caused highly effective tumor growth suppression. These findings fit with the previously published in vitro studies demonstrating impaired cell and colony growth with ITIH5 [3,7,8]. Since slow-growing ITIH5 re-expressing tumors are characterized by low vessel density and mature blood vessels, while fast-growing ITIH5 deficient tumors show a dense immature network of blood vessels, the differences in blood vessel density and maturation between the two groups are consistent with their differences in tumor growth. However, a slight effect on the mechanisms of neo-vessel formation could be shown in vivo, which, importantly, may further support the repression of tumor cell growth being primarily affected by the intrinsic features of cancer cells, such as altered cell cycle control or apoptotic mechanisms, as previously demonstrated [7]. Thus our in vivo data complement the already existing view of *ITIH5* as a potent tumor suppressor gene involved in regulating various hallmarks of cancers, i.e., tumor progression [7,8] and tumor growth, as both functions may be important for the early stages of tumor development.

### 3.2. ITIH5 VIT Domain Applied Extracellularly Is Sufficient to Mediate Tumor Cell Growth

Next, we provided evidence that the VIT domain of the ITIH5 protein administered extracellularly to cancer cells in vitro may make an important contribution to the inhibition of tumor cell growth observed in vivo. We are aware that mechanistic details cannot be given yet; however, the in vitro response of diverse cancer cell lines to treatment with the truncated ITIH5-derived protein fragments (ITIH5^161aa^ and ITIH5^681aa^) suggested a highly conserved and ITIH5 (signaling)-specific mode of action: (1) The ITIH5^681aa^ fragment covering both the VIT and the vWA domain did not affect growth of benign mammary cells nor that of MDA-MB-231 single cell clones with strong overexpression of the full-length *ITIH5* gene. However, in ITIH5-deficient malignant cells such as wild-type MDA-MB-231 or T47D, reflecting distinct molecular breast cancer subtypes (basal (TNBC) and luminal), ITIH5^681aa^ suppressed cell and colony growth in vitro. (2) ITIH5^681aa^ as well as the downsized ITIH5^161aa^ fragment caused growth inhibition in a dose-dependent manner in these cancer cells, in which the suppressive impact of the full-length ITIH5 has been confirmed previously, i.e., in breast cancer [2,7], bladder cancer [3] and adenocarcinomas of the lung (NSCLC) [5]. Apoptotic mechanisms were not affected in breast cancer cells. (3) The dose-dependent survival curves of cancer cell lines treated either with ITIH5^681aa^ or ITIH5^161aa^ were highly similar when classifying cells into responders (e.g., MDA-MB-231) and non-responders (H157), i.e., the truncated ITIH5^161aa^ polypeptide comprising 161 aa in size simulated the growth-inhibitory effects of the *N*-terminal ITIH5t protein (ITIH5^681aa^), suggesting the substantial role of VIT. Since the growth inhibition of MDA-MB-231 ΔpBK-ITIH5 clones known to re-express the full-length ITIH5 protein could not be further triggered, the responsiveness of cancer cells according to the ITIH5 polypeptides applied may depend on the availability of cell-type-specific factors to restore ITIH5’s tumor suppressive pathway.

Mechanistically, the recombinant polypeptides derived from ITIH5 did not contain the conserved cleavage site, and thus the growth inhibitory impact should reflect a mechanism independent of HA-binding. In light of the dynamics and efficacy of the drug response curves mediated by the truncated ITIH5 polypeptides, which were limited to a maximum of 50% growth inhibition, a saturation point due to protein–protein interaction could be suggested. In addition, our data suggest an ECM-associated function of truncated ITIH5 proteins, since both ITIH5^681aa^ and ITIH5^161aa^, whose amino acids include the endogenous signal peptide, were clearly secreted. So far, no indication of internalization by cells was found after proteins were administered in vitro. However, data for the ITIH5^161aa^ polypeptide remain elusive. Different *pK_a_* values as well as the range of different molecular weights of ITIH5-derived protein fragments (both ITIH5^681aa^ and ITIH5^161aa^) indicate intensive post-translational modifications, which are known to achieve the full biological activity of ECM proteins [36]. However, it is obvious that future studies are required to consider further mechanisms which may be causative for the different suppressive effects observed, including the putative intracellular function recently postulated [35]. Young et al. showed that a secretion-deficient ITIH5 protein lacking the endogenous signal peptide could be involved in the morphological changes of pancreatic cancer cells and suppression of metastasis of these cells [35]. Bearing in mind that ITIH5 did not impair the growth of pancreatic cancer cells [10], intracellularly acting ITIH5 proteins might be particularly responsible for the changes in cell plasticity. Interestingly, Liu and colleagues presented Kruppel-like factor 4 (KLF4) as an intracellular binding partner of ITIH5 whose expression is induced by p53, while ITIH5′s interaction with KLF4 fosters the transcriptional inactivation of KLF4 in melanoma cells [9]. KLF4 has been shown to interact directly with Oct4 and Sox2 which are critical for somatic cell reprogramming [37], while in breast cancer, KLF4 may have a potent oncogenic role in tumorigenesis by maintaining stem cell-like features and by promoting cell migration and invasion [38]. However, data providing details of the ITIH5 binding sites involved in intracellular KLF4 interactions are missing and must be deciphered in future studies.

In summary, we revealed, for the first time, the specific tumor-suppressive function of the ITIH5 VIT domain, but it seems difficult to assess whether the VIT sequences of heavy chains in general mediate tumor growth inhibition. We compared the homology of the VIT sequence of heavy chains 1–4 with that of ITIH5, determining a range between 26% and 37% only; thus, the ITIH5 VIT domain and its functional role in tumor suppression might indeed be heavy-chain-specific. Considering that it is known that ITIH5 contains all the structural features found in ITIH1-3 (i.e., the VIT, vWA and C-terminal heavy chain domain), its expression pattern clearly differs from that of other heavy chains. Unlike ITIH1-4, ITIH5 is rarely expressed in the liver but is strongly expressed in the epithelial tissues of various organs such as the mammary gland [39] or urothelial layers [3]. Moreover, *ITIH5* is the only member of the ITI genes that are inactivated by epigenetic mechanisms which substantially trigger tumor development, plasticity and metastasis. Since Weidle and colleagues proposed the vision of an ITI-derived protein approach suitable for developing novel anticancer therapies [20], our study may present a way to restore the tumor-suppressive function of ITIH5 in a much smaller polypeptide. Thus, truncated polypeptides, especially ITIH5^161aa^, which covers only the VIT domain, may serve as biologicals to reactivate ITIH5-associated tumor-suppressive pathways in cancer cells, for instance, in aggressive TNBC, for which most experimental data have been given [7,8], helping to effectively impair tumors’ growth and potential spreading.

## 4. Materials and Methods

### 4.1. Wild-Type and Genetically Modified Cell Lines

Wild-type breast (MDA-MB-231, T47D, BT20, MCF10A) and lung cancer cell lines (H157, A549) were originally obtained from the American Type Culture Collection (ATCC, Manassas, VA, USA). MDA-MB-231-derived single-cell clones (#1, #7, #12) with either stable ITIH5 overexpression (ΔpBK-ITIH5 clones) or a complete lack of ITIH5 expression (ΔpBK-mock clones) were generated and have been characterized previously [7]. SCaBER, a basal bladder cancer cell line with squamous features, was kindly provided by Dr. Michèlle Hoffmann (Düsseldorf University Hospital, Germany). All cell lines were known to lack *ITIH5* mRNA expression (see Figure S2 [40,41]) and were regularly tested for mycoplasma infection using the PCR-based Venor^®^ GeM Mycoplasma Detection Kit (Minerva Biolabs, Berlin, Germany) prior to the experiments. The genetic status of the TP53 gene was assessed by using the Cancer Cell Line Encyclopedia of the cBioportal database [40,41].

### 4.2. Animals

Overall, n = 18 female BALB/c^nu/nu^ mice were purchased from Charles River Laboratories International (Wilmington, MA). The procedures and experiments were conducted in accordance with the German federal law regarding the protection of animals. All protocols were approved (AZ 87-51.04.2010.A226) by the administration of the Landesamt für Umwelt, Natur und Verbraucherschutz (LANUV, Recklinghausen, Germany).

### 4.3. Construction and Generation of Truncated Recombinant ITIH5-Derived Proteins

ITIH5-derived cDNA sequences were cloned into a vector (pMS-L-A-IV vector), provided by the Fraunhofer-IME (Institute for Molecular Biology and Applied Ecology, Aachen, Germany). The vector construct contains a CMV promoter and a multi-cloning site, including the restriction sites NheI and NotI. The DNA sequences of the truncated ITIH5 polypeptides were amplified by PCR using the pBK-CMV plasmid which includes the full-length ITIH5 cDNA, used for in vitro modeling in previous studies (see [2,7]) based on the following oligonucleotide primers: ITIH5^681aa^ forward and ITIH5^161aa^ VIT ITIH5 forward primer: 5-GCGC**GCTAGC**ATGCTCCTGCTGCTGGGG-3, bold letters: NheI consensus site; ITIH5^681aa^ reverse primer: 5-GCGC**GCGGCCGC**ATCACCACCATCCACTGATGTTTTAG-3, bold letters: NotI consensus site; and ITIH5^161aa^ reverse: 5-GCGC**GCGGCCGC**AAGCTCCTCATAACTCAGGAA-3, bold letters: NotI consensus site. After NheI/NotI digestion, the PCR fragments were cloned into the pMS-L-A-IV expression vector and digested with the same restriction enzymes. The resulting recombinant construct was verified by DNA sequence analysis.

The cloned vector constructs were transferred into the nuclei of human HEK293T cells via Fugene (Promega, Madison, WI, USA) and expanded under zeocin conditions (700 µg/mL; Invitrogen, San Diego, CA, USA). After 2 to 4 weeks of culturing (37 °C, 5% CO_2_, 95% relative humidity), the supernatant of transfected HEK293T cells was harvested. Based on the principle of affinity chromatography, the recombinant ITIH5-derived proteins present in the supernatant of HEK293T cells were purified by using Ni-NTA agarose beads (Qiagen, Hilden, Germany). In brief: the supernatant was incubated (2 h, room temperature) with a 4× incubation buffer (200 mM NaH_2_PO_4_ (pH 8.0), 1.2 M NaCl, 40 mM imidazol (pH 7.0)) and Ni-NTA agarose (50%)). After centrifugation, the Ni-NTA pellet was washed with 1× incubation buffer and finally re-suspended in an elution buffer (50 mM NaH_2_PO_4_ (pH 8.0), 300 mM NaCl, 250 mM imidazol (pH 7.0)). To remove the imidazol for subsequent functional in vitro analyses, the isolated peptides were further enriched and rebuffered in PBS via Vivaspin 6 columns (Sartorius Stedim, Göttingen, Germany) according to the manufacturer’s instructions. Western blot analyses were used to confirm the purity and amount of the corresponding ITIH5^681aa^ and ITIH5^161aa^ protein batches.

### 4.4. Nucleic Acid Extraction and Reverse Transcription PCR

RNA was isolated using TRIzol^TM^ reagent (Thermo Fisher Scientific), and 1 µg of RNA was used for cDNA reverse transcription by the Promega Kit A3500 according to the manufacturer’s instructions (Promega, Mannheim, Germany).

### 4.5. Real-Time qPCR

*ITIH5* mRNA expression levels in growing tumors of the mouse model were confirmed by real-time PCR using the SYBR-Green PCR mix (Bio-Rad Laboratories, Munich, Germany) in an iCycler IQ5 (Bio-Rad Laboratories), as previously specified [7].

### 4.6. Two-Dimensional (2D) Gel Electrophoresis (2-DIGE)

Extracted proteins were mixed with a rehydration buffer (7 mM urea, 2 M thiourea, 2% *w*/*v* CHAPS, 1% DTT, 1% IPG buffer (pH 3.11 NL) (GE Healthcare, Bucks, UK), 0.002% bromphenol blue) to give a total volume of 450 μL. Rehydration, isoelectric focusing and gel electrophoresis were performed as described by Labbus et al. [42].

### 4.7. Coomassie Blue Staining

Gels were fixed with 45% (*v*/*v*) methanol, 5% (*v*/*v*) acetic acid (C_2_H_4_O_2_) and 50% (*v*/*v*) H_2_O for ≥30 min. After removal of the acetic acid by three subsequent washing steps with H_2_O, staining was completed by applying a Coomassie solution for 15 min. The reaction was stopped with 1% (*v*/*v*) acetic acid.

### 4.8. Protein Verification by Western Blotting

Western blot analysis to confirm the full-length ITIH5, ITIH5^681aa^ and ITIH5^161aa^ was performed as recently described [8], with slight modifications for the truncated polypeptides. After blocking in Tris-buffered saline containing 0.01% Tween-20 (TBS-T) and 5% non-fat dry milk (Roth, Karlsruhe, Germany), blots were probed with the primary antibody Penta-His-tag (C-Term) ((P-21315); 1:1000, Invitrogen, Carlsbad, CA, USA) in a blocking solution buffer, washed and incubated with goat anti-mouse 1:5000 ((P0447); Dako, Glostrup, Dänemark) secondary peroxidase-conjugated antibodies. Antibody detection was accomplished with Pierce ECL Western Blotting Substrate (Thermo Scientific, Rockford, IL, USA). Equal protein loading was monitored by using a β-actin-specific antibody.

### 4.9. Protein Identification by MALDI-TOF MS/MS

Selected spots of 2-DIGE Coomassie-stained gels were digested. Trypsin digestion and protein identification using MALDI-TOF MS and MS/MS were carried out as previously described [40]. The MASCOT^TM^ database was used (Matrix Science Ltd., London, UK [43]).

### 4.10. Cytotoxicity Assay

To exclude any non-specific effects of the truncated ITIH5 proteins on the integrity of membranes, lactate dehydrogenase (LDH) activity was determined under recombinant ITIH5 protein treatment of cancer cells (1 × 10^4^ cells / well; 96-well plates) by using the Cytotoxicity Detection Kit (Roche Diagnostics, Mannheim, Germany) according to the manufacturer’s instructions, including “low” and “high” controls. Optical density was measured with an ELISA-Reader Infinite M200 Tecan (Bio-Rad Laboratories, Munich, Germany). Each assay was independently performed at least three times. Cytotoxicity was then calculated as follows: (Sample − low control)/(high control − low control) × 100.

### 4.11. Short-Term Drug Response Assay

For each dose, 1 × 10^4^ cells/well were grown in triplicate in a 6-well-plate. Next, 24 h after seeding, recombinant ITIH5 proteins (either ITIH5^681aa^ or ITIH5^1681aa^) were added to each well in distinct concentrations (i.e., 0.25, 0.5, 0.75 and 1.0 μg/mL) and incubated for a further 72 h. Cells were then harvested, and the number of viable cells was determined using the automated CASY 1 system (Schärfe System, Reutlingen, Germany). PBS was added as a negative control (0.0 µg/mL protein). Scrambled proteins (1 µg/mL) which were isolated from the supernatant of wild-type HEK293T cells were added to exclude host-dependent effects on the growth of cancer cells. All experiments were independently performed in triplicate.

### 4.12. Clonogenic Survival Assay

For each dose, 1000 cells were seeded in a 6-well plate in triplicate. Recombinant ITIH5 proteins (either ITIH5^681aa^ or ITIH5^1681aa^) were added in various concentrations (i.e., 0.05, 0.1 and 1.0 μg/mL) to the cells, analogous to the short-term drug response assay. PBS was added as a negative control. Treated cells were incubated at 37 °C for 10 days, while renewing the medium including the recombinant ITIH5 proteins every 2 to 3 days. Formation of colonies was regularly monitored under a light microscope. Subsequently, wells were washed with PBS, fixed and stained (30 min) with 1 mL of a 0.1% formalin-containing crystal violet solution. Pictures of grown colonies were taken, and the relative colony growth rates were densitometrically calculated by using ImageJ software (National Institute of Health, Bethesda, MD, USA). Each assay was independently performed at least 3 times.

### 4.13. Apoptosis Assay

The activity of the effector caspases 3 and 7 in breast cancer cells was analyzed by using the Apo-One^®^ Homogeneous Caspase-3/7 Assay (Promega, Mannheim, Germany) according to the manufacturer’s instructions. MDA-MB-231 cells (1.5 × 10^4^) were plated into 96-well plates and incubated overnight (20% O_2_, 5%CO_2_, 37 °C). Afterwards, ITIH5^681aa^ and ITIH5^161aa^ (1 μg/mL) were applied in triplicate and incubated for 24 and 48 h, respectively. Fluorescence intensity was quantified by using an ELISA plate reader (excitation: λ = 485 nm; emission: λ = 577 nm).

### 4.14. Wound Healing Assay

To assess the capacity of breast cancer cells to close a wounded area in vitro, a monolayer scratch wound assay was performed as previously specified [3], with slight modifications: ITIH5^681aa^ and ITIH5^161aa^ protein (1 μg/mL) was applied 24 h before scratching the cell layer.

### 4.15. In Vivo Mouse Model

A xenograft tumorigenesis model was conducted to assess the impact of ITIH5 on (breast) cancer cell growth in vivo. Five-week-old female BALB/c^nu/nu^ nude mice were used to orthotopically inject human MDA-MB-231 breast cancer cells (1 × 10^6^ in 100 µL PBS) into the right posterior mammary fat pad. Nine mice were either injected with the test group (ITIH5 clone #7) or the control group (empty vector clone #1). After injection and growth of the tumors, the tumors were measured twice a week with a caliper. The volume (V) of tumors was determined by the formula V = 0.52 × D × d^2^ (D: largest tumor diameter; d: smallest tumor diameter) according to Tugues et al. [44]. The animals were continuously monitored for irregularities in behavior or signs of pain and suffering.

### 4.16. Fluorescence Imaging

Staining of paraffin-embedded tumor sections was performed with CD31 (Dianova, Hamburg, Germany) and α-SMA-biotin (PROGEN Biotechnik, Heidelberg, Germany) antibodies. Nuclei were counterstained using Hoechst nuclear dye. Fluorescence microscopy was performed using an Axio Imager M2 light microscope (Carl Zeiss Microimaging, Göttingen, Germany) and photographed with an AxioCam MRm VErsion 3 high-resolution camera (Carl Zeiss, Jena, Germany). Images were analyzed and quantified using ImageJ 1.44p (National Institutes of Health, Bethesda, MD, USA). The determination of filled CD31 area fractions was described previously [21].

### 4.17. µCT Imaging

In order to visualize and study the angiogenesis of tumors grown in our in vivo model, mice were screened by using non-invasive in vivo 3D µCT imaging (TomoScope 30s Duo CT Imaging, Erlangen, Germany). Animals were anesthetized by applying 1.5 Vol% isoflurane in 100% oxygen. To visualize the vascular structures, the iodine-containing contrast medium eXIA 1600^®^ (Binitio Biomedical, Ottawa, Germany) was administered to the animals via a tail vein catheter. Imaging was performed using dual-energy scans at 41 and 65 kV (at 0.5 mA and 1 mA) for each mouse, leading to the calculation of 2880 images (size: 1032 × 1024 pixels) over a scanning period of 6 minutes under constant rotation. The VOXEL-MAN program system (Feldkamp type; CT imaging, Erlangen, Germany) was used to reconstruct the images, which were then analyzed by AMIDE software [45]. Finally, the 3D architecture of the images was completed by using the Imalytics research workstation 3.0 beta (Philips Research, Aachen), allowing interactive image segmentation for calculating both the tumor/organ volume and the relative blood volume (rBV). rBV was determined by calculating the radiopaque value (Hounsfield units) of the segmented tumor and a reference blood vessel (abdominal part of the aorta) before (0% rBV) and after (100% rBV) injection of the contrast agent as follows: rBV [%] = tumor (after contrast medium) − tumor (prior contrast medium)/blood vessel (after contrast agent) − blood vessel (before contrast agent) × 100.

### 4.18. Statistical Data Acquisition

Two-sided *p*-values less than 0.05 were considered significant. In order to compare the two groups, the non-parametric Mann–Whitney U-test was implemented, whereas in the case of more than two groups, Dunn’s multiple comparison test was used. Correlation analysis was performed by calculating the non-parametric Spearman’s rank correlation coefficient.

## 5. Conclusions

Since extracellularly administered ITIH5-derived polypeptides covering the VIT domain could mimic the growth-inhibitory effects of the native ITIH5 protein in a set of different cancer cell lines, our data suggest a substantial role of the VIT domain in restoring tumor suppressive function of ITIH5. Given that, truncated ITIH5 polypeptides, especially ITIH5^161aa^ may constitute the basis for developing of biologicals to treat human cancers lacking endogenous ITIH5 expression, thus helping to effectively block tumorigenesis.

## Figures and Tables

**Table 1:** Alignment statistics for the ITIH5-derived VIT sequence (ITIH5^161aa^).

Aligned Sequences	Expected	Method	Identities	Positives	Gaps
Matching ITIH5 VIT (56–161 aa)–ITIH1 VIT (58–166 aa)	2 × 10^−10^	Compositional matrix adjustment	29/111 (26%)	55/111 (49%)	7/111 (6%)
Matching ITIH5 VIT (51–114 aa)–ITIH2 VIT (72–135 aa)	7 × 10^−14^	Compositional matrix adjustment	23/64 (36%)	41/64 (64%)	0/64 (0%)
Matching ITIH5 VIT (56–161 aa)-ITIH3 VIT (50–158 aa)	6 × 10^−16^	Compositional matrix adjustment	34/109 (31%)	56/109 (51%)	3/109 (2%)
Matching ITIH5 VIT (54–161 aa)–ITIH3 VIT (38–148 aa)	1 × 10^−17^	Compositional matrix adjustment	41/112 (37%)	59/112 (52%)	5/112 (4%)

## Data Availability

The data that support the findings of this study are available from the corresponding author upon reasonable request.

## Supplementary Materials

The following supporting information can be downloaded at: https://www.mdpi.com/article/10.3390/cancers14030488/s1. The datasets supporting the conclusions of this article are included within the article and its additional files. Figure S1: Confirmation of ITIH5^681aa^ by 2D-SDS-PAGE and mass spectrometric PMF assays (MALDI-TOF). Figure S2: Illustration of the relative endogenous ITIH5 mRNA expression, TP53 mutations and ITIH5t responsiveness of malignant cell lines selected for functional analysis in this study compared with ITIH5-overexpressing skin cancer cell lines. File S1: Original Images of Western Blot figures.
