# Supplementary material for: ITIH5-Derived Polypeptides Covering the VIT Domain Suppress the Growth of Human Cancer Cells In Vitro
Source: Cancers (Basel). 2022 Jan 19;14(3):488. doi: 10.3390/cancers14030488 (PMC8833355; doi:10.3390/cancers14030488)
Supplement: Supplementary file 1 [file cancers-14-00488-s001.zip › Supplementary Figure S1.pdf]

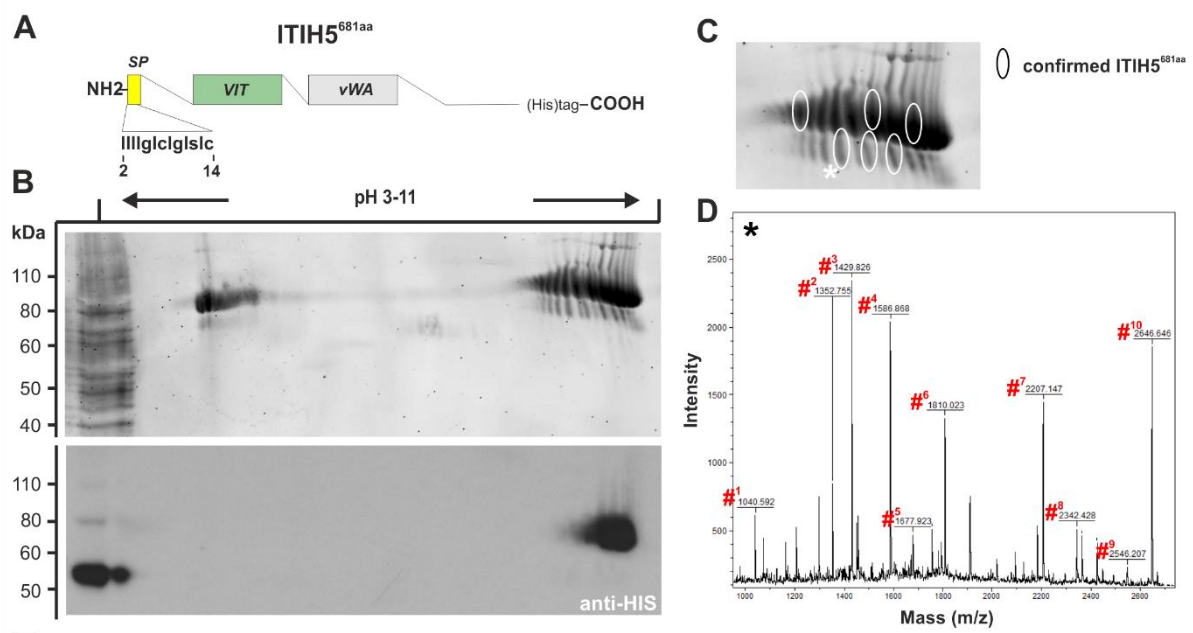

E

1) 1040.5920 = RFSIIGFSNRI

2) 1352.7550 = KDHLISVTPDSIRD

3) 1429.8260 = RIAQNGILGDFIIRY

4) 1586.8680 = KAAFFLSYEELLQRR

5) 1677.9230 = KLDHLHVEVTASNSKKF

6) 1810.0230 = KDALFTILHDLRPQDRF

7) 2207.1470 = RVHEEEDAGSQLIGFYDEIRT

8) 2342.4280 = RSVSLIVFLTDGKPTVGETHTLKI

9) 2546.2070 = RMDGLEEAHGMSAAMGPEPVVQSVRG

10) 2646.6460 = RLSVDVNILESAGIASLEVLPLHNSRQ

Figure S1: ITIH5^{681aa} purified from HEK supernatant and analyzed by 2D-SDS-Page analyses. **(A)** Scheme of the truncated ITIH5 protein including the VIT and vWA domains. SP: signal peptide; VIT: Vault Inter-Trypsin domain; vWA: von Willebrand factor type-A domain according to EMBOSS 6.3.1:sigcleave, Ensembl.org, and PFAM.sanger.ac.uk. **(B)** Coomassie-stained 2D gel (upper image) of the supernatant of cultured human HEK cells after transfection with cloned pMS-L-A-IV vectors. Lower image shows the corresponding anti-(His)tag western blot analysis in a pH range of 3-11 and a mass range of 40-120 kDa. **(C)** Confirming the ITIH5^{681aa} by mass spectrometric PMF assays (MALDI-TOF). Shown gel highlights regions included in the PMF analysis (white bordered). The following data are based on the spot marked with an asterisk. **(D)** Exemplary PMF spectrum of a trypsin-digested spot. The signals (here: shown for the highest signal values #1-#10) could be assigned to the mass of corresponding peptide sequences **(E)** of the ITIH5^{681aa} fragment with a score of 157. Used PMF parameters were selected as follows: *Organism:* Homo sapiens; *Enzyme:* Trypsin; variable modification: oxidation (M); mass value: monoisotopic; protein mass: Unrestricted; Peptide mass tolerance limit: ±0.8 Da; Number of mass value searches: 13.

.
