# Supplementary material for: ITIH5-Derived Polypeptides Covering the VIT Domain Suppress the Growth of Human Cancer Cells In Vitro
Source: Cancers (Basel). 2022 Jan 19;14(3):488. doi: 10.3390/cancers14030488 (PMC8833355; doi:10.3390/cancers14030488)
Supplement: Supplementary file 1 [file cancers-14-00488-s001.zip › Supplementary Figure S2.pdf]

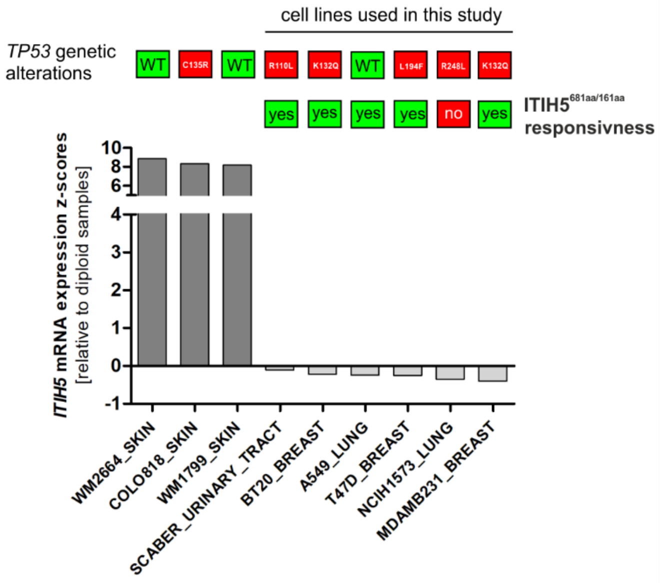

Figure S2: Illustration of the relative endogenous *ITIH5* mRNA expression (see Cancer Cell Line Encyclopedia of the cBioportal data base [40,41]) of malignant cell lines (light grey bars) selected for functional analyses in this study compared to ITIH5 high-expressing cancer cell lines of the skin (dark grey bars). The mutational status of TP53 is indicated (green: wildtype (WT) and red: mutation, single nucleotide polymorphism (SNP)). The responsiveness of malignant cells towards application of truncated ITIH5 polypeptides ($ITIH5^{161aa}$ and $ITIH5^{681aa}$) is indicated (green: responder cell line; red: non-responder cell line).
