# Supplementary figures and images for: ITIH5-Derived Polypeptides Covering the VIT Domain Suppress the Growth of Human Cancer Cells In Vitro
Source: Cancers (Basel). 2022 Jan 19;14(3):488. doi: 10.3390/cancers14030488 (PMC8833355; doi:10.3390/cancers14030488)

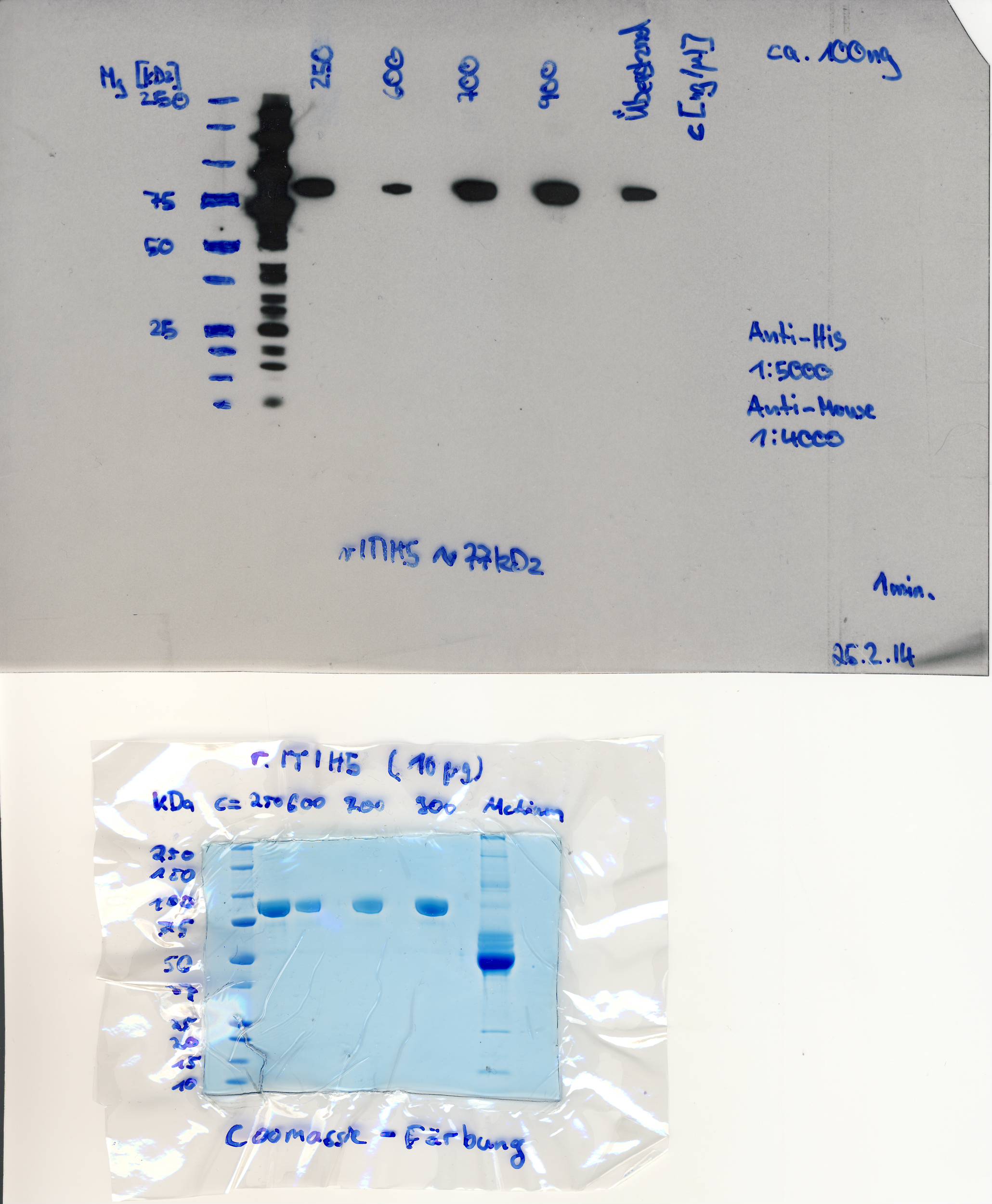

Supplement: Supplementary file 1 [file cancers-14-00488-s001.zip › rek. ITIH5.JPG]

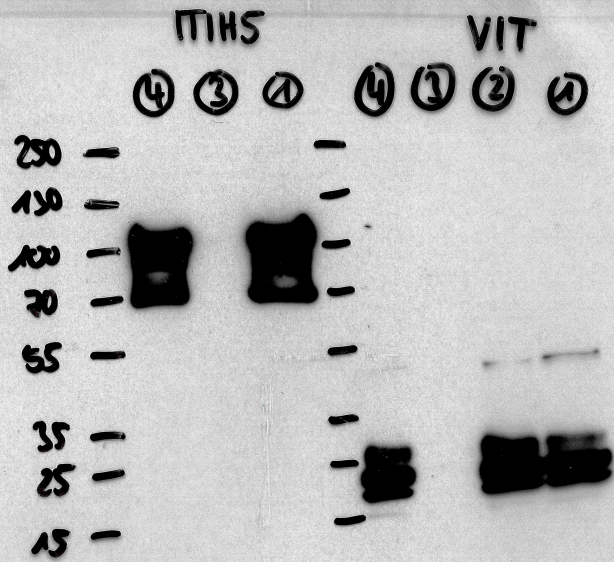

Supplement: Supplementary file 1 [file cancers-14-00488-s001.zip › Scan_2021-08-23 15-11-28.pdf]

5min

Control

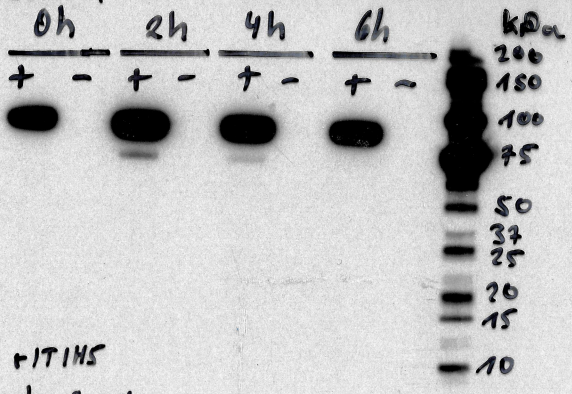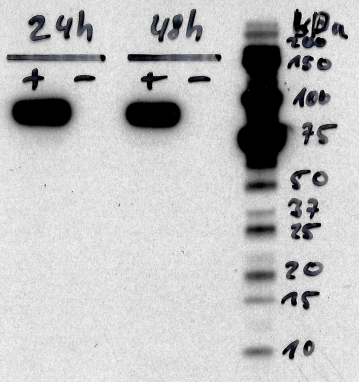

+ = FITINS  
- = olive Engate

Supplement: Supplementary file 1 [file cancers-14-00488-s001.zip › Scan_2021-08-30 09-27-37.pdf]
